# Testing scenarios for using telepresence robots in healthcare settings

**DOI:** 10.1016/j.csbj.2024.01.004

**Published:** 2024-01-17

**Authors:** Janika Leoste, Kadri Strömberg-Järvis, Tarmo Robal, Kristel Marmor, Katrin Kangur, Anne-Mari Rebane

**Affiliations:** a Tallinn University, Narva rd 25, 10120 Tallinn, Estonia; b Tallinn University of Technology, Ehitajate tee 5, 19086 Tallinn, Estonia; c Tallinn Health Care College, Kännu 67, 13418 Tallinn, Eestonia

**Keywords:** Telepresence robots, Healthcare, Telehealth, Ageing population

## Abstract

The ageing global population puts heavy pressure on healthcare systems everywhere. Addressing ageing-related chronic conditions requires employment of novel innovative solutions. Telehealth technologies, including telepresence robots (TPRs), are being rapidly developed to provide healthcare services efficiently wherever needed. This article explores the role of TPRs in addressing the challenges of providing healthcare to an ageing population, emphasizing their potential advantages and drawbacks. Employing an exploratory research approach with qualitative data collection techniques, we tested three TPR usage scenarios in simulated healthcare settings: anamnesis, measurements, and falls and frailty. The study employed a non-random purposive sample comprising 25 participants, and was conducted at a medical facility in June 2023. The findings suggest that TPRs offer promising solutions for healthcare professionals and patients, especially in scenarios when physical presence is impossible or physical isolation is required to prevent contagion. However, the technology is not yet ready to substitute fully human medical workers, potentially causing patient reluctance and emphasizing the need for patient-centered approaches to technology adoption. In addition, more studies are needed to address ethical, privacy, and scalability concerns.

## Graphical Abstract

**Figure:**
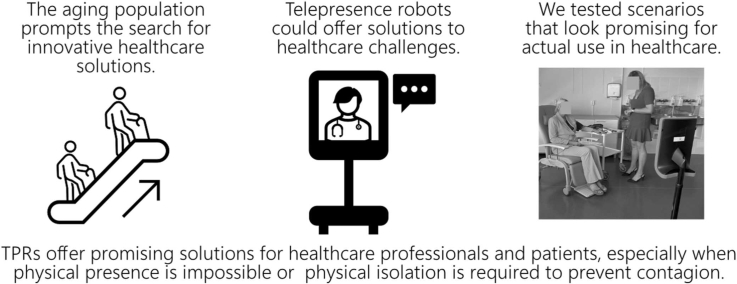

## 1 Introduction

The population is ageing globally, leading to profound implications for healthcare systems. In comparison to the past century, life expectancy has nearly doubled [1]. Many developed and developing countries are contending with a significant increase in the number of elderly individuals. This demographic shift poses both health and economic challenges, as the prevalence of chronic diseases, often associated with ageing, continues to rise [2], [3]. Chronic conditions such as cardiovascular issues, stroke, cancer, osteoarthritis, and dementia impose a substantial burden on healthcare systems and budgets [4]. The escalating costs of healthcare delivery and drug development, coupled with the growing occurrence of chronic and lifestyle-related disorders, emphasize the pressing need for innovative strategies in healthcare [5] healthcare challenges. The burden on healthcare systems is further compounded by the desire of empowered patients to participate actively in their treatment decisions and the demand for more convenient and value-added healthcare services [5]. Given the stakes in health and well-being, addressing these challenges becomes paramount.

One promising solution in the realm of healthcare innovation is telehealth, which encompasses various remote healthcare delivery methods, including telehealth, mobile health, and electronic health [6]. Telehealth holds the potential to bridge the gap in healthcare access, especially for elderly individuals and those in remote or underserved areas. In light of the increasing prevalence of chronic conditions and the limitations of in-person healthcare delivery, telehealth holds the potential to become a critical tool for providing quality care and improving health outcomes [7], [8]. A recent addition to telehealth technologies is telepresence robotics. Telepresence robots (TPRs) are robots equipped with cameras, microphones and various sensors that allow their users to establish spatial and social presence over distance or in situations where direct biological presence is either impossible or undesired [9].

This paper explores the role of TPRs as a solution to the challenges of providing healthcare to an ageing population, with a focus on its potential advantages and disadvantages, access to care, and the impact on healthy ageing. Our aim is to investigate the potential of TPR applicability in healthcare through developing and evaluating different scenarios for medical settings. In the following sections we: (a) present a short overview of the relevant literature about the use of TPRs in healthcare environments; (b) using the ideas and suggestions from the overview and from medical experts, we design three scenarios for using TPRs in healthcare; (c) test the scenarios in a medical school’s laboratory; and (d) based on the feedback from the participants, evaluate the scenarios. These steps represent a pilot experiment that must precede to the use of TPRs in real medical settings.

## 2 Related work

### 2.1 The concepts of telehealth and telepresence robot

*Telehealth***,** also known as telemedicine, is a contemporary approach to healthcare delivery that uses electronic communication for the transmission of medical information with the primary objective of enhancing patient well-being [10]. This innovative method of healthcare provision can be broadly categorized into two main types: synchronous and asynchronous telehealth [7]:

*Synchronous telehealth*, often-referred to as real-time telehealth, revolves around the utilization of audiovisual technology that enables individuals to engage in immediate and real-time interactions through, for example, a video conferencing platform. To participate effectively in this form of telehealth, users require essential hardware components, including a video camera, sound system, computer screen, and a reliable high-speed internet connection for seamless data transmission between locations. Consequently, synchronous telehealth heavily relies on the availability of robust video conferencing infrastructure, primarily accessible at institutional levels.

A cost-effective alternative, *asynchronous telehealth*, also known as store-and-forward telehealth, operates without the necessity for synchronized interactions between parties. This type of telehealth involves collection of digital data, such as electrocardiograms, spirometry results, or radiology images, in one location, which is subsequently transmitted to another location for assessment by healthcare professionals. The technology underpinning asynchronous telehealth encompasses equipment capable of capturing, storing, and facilitating transfer of digital data to remote locations.

In the context of the recent pandemic, telehealth has emerged as a critical tool in delivering healthcare services over distances, although the availability of high-quality evidence-based trials remains limited [11]. Telehealth solutions have proven to be both viable and acceptable in providing care to older adults in Long-Term Care Facilities (LTCFs), even among those facing sensory impairments like hearing or visual loss [11].

A recent addition to telehealth technologies is the use of telepresence robots (TPRs) – an emerging technology in the field of remote communication and collaboration. At its core, telepresence, as defined by El-Gayar et al. [12], encapsulates the sensation of being wholly immersed in a location far removed from one's physical presence, effectively constructing a virtual environment that mirrors genuine experiences for the operator of the robot. TPRs are remotely controlled mobile devices on wheels, adorned with various features, including cameras, speakers, microphones, a screen, sensor-assisted motion control, and other interactive components, designed to facilitate seamless remote communication and collaboration. This allows remote users to assume control via computers, tablets, or smartphones while simultaneously immersing themselves in the robot's on-site surroundings displayed on a screen. Moreover, the user's face is projected onto the robot's screen, permitting face-to-face interactions with individuals at the robot's physical location, essentially forging a form of remote telepresence [13]. Compared to videoconferencing, a TPR allows the remote person to choose and change their location in the room, and focus their attention on the persons or objects at different locations of the room.

The use of TPRs has expanded across diverse fields, including medicine, elderly care, office work, museums, industry, and education [14]. The appeal of TPRs is underpinned by their cost-effectiveness, when compared to real physical presence, timesaving capabilities, and their capacity to facilitate enhanced communication and social presence [15]. In essence, TPRs break down the barriers imposed by distance, allowing individuals to participate in remote events, bridging the gap for those constrained by chronic illnesses, medical conditions, or disabilities that make face-to-face interactions impractical. As such, TPRs serve as a transformative tool, revolutionizing remote communication and collaboration, adding value to the delivery of various services, particularly in domains like healthcare, education, and culture [13], [16], [17].

Despite their remarkable potential, TPRs are still considered a novelty, meant for early adopters, and the social, psychological, and pedagogical dimensions of interactions with these robots remain areas warranting further exploration [18], [19], [20], [21].

### 2.2 Potential usage scenarios for TPRs in healthcare

Numerous usage scenarios have been proposed and tested for implementing TPRs within the telehealth context. A recurring theme is the need to address the increased workload of nurses and doctors due to a growing number of elderly patients while preserving the patients' sense of social inclusion. Existing literature indicates that constant supervision can be advantageous for elderly patients, enabling medical personnel to respond promptly to health disorders or risk factors, such as falling or congestive heart failure, which could lead to serious or fatal injuries or serve as initial signs of impending health deterioration [22], [23], [24], [25]. In particular, the use of video monitoring has been recommended to enhance reaction time while reducing the workload of dedicated medical personnel [25]. Nonetheless, this approach comes with its own set of drawbacks, notably concerning the potential for privacy breaches or perceived social isolation [26], [27]. Of these, perceived social isolation, or insufficient social presence, could have direct consequences on the patient’s health due to its negative influence on patients' physical, mental, and emotional health, potentially increasing their reliance on health services [28]. Social presence is the feeling of being connected to others in a virtual or mediated environment, resembling the sense of physical presence [13]. Studies indicate that TPRs are more efficient in reducing social isolation; that is to say, they support better social presence of remote individuals compared to video conferencing-based solutions [29], [30]. The reasoning above suggests that bringing a TPR to the same physical room with an elderly patient could serve as a non-intrusive telehealth tool supporting the patient’s feeling of social belonging, but also for monitoring the patient’s health status, administering medications or measuring the patient's medical parameters (e.g., pulse, blood pressure or oxygen saturation levels), or enabling discussions between the patient and their doctor or family members. Still, little knowledge is available on the applicability of TPR’s for medicine and healthcare. We continue by exploring three case studies on telehealth [31], [32], [33] that we used as inspiration for developing the scripts for our experiment (Section 3.2).

First, Wong et al. [31] examined a simulation-training program that focuses on enhancing inter-professional collaboration and the utilization of telehealth technology in health studies. In their study, pharmacy and nursing students were engaged in collaborative exercises using telepresence robots. The program's core objectives included improving role understanding, communication, team collaboration, and coaching skills for patient interaction with telehealth technology. In their study, faculty members designed and validated two simulation scripts, crafted to provide students with opportunities to develop telehealth skills, exercise professional roles, and collaborate effectively during patient care encounters for the development of comprehensive treatment plans. In both cases, students operated a telepresence robot to conduct a focused patient assessment, discuss their findings, and collaborate for formulating a well-informed plan of care. Quantitative findings demonstrated significant improvements in communication, teamwork, and patient involvement. Qualitative feedback highlighted the program's realism and identified areas for improvement, notably in audio quality and orientation of TPRs for novice students. The authors emphasize the importance of clear guidelines, scripted questions, smaller group sizes, focus group sessions, direct observation, and the shift to a fully online/telehealth format to prepare students for evolving healthcare practices.

Second, Isabet et al. [32] offer an extensive review of experiments involving TPRs in the context of older adults, both before and during the COVID-19 pandemic. The primary aim of the study was to assess the usability, acceptance, and potential benefits of these robots in enhancing the well-being of older adults. Their research found that, in general, older adults had a positive response to using TPRs, finding them engaging and experiencing minimal negative side effects. Telepresence robots were considered useful tools for maintaining social connections and reducing loneliness among older adults. In addition, the authors also suggest that some of the TPRs have features that could facilitate the daily life of elderly patients such as reminders for appointments and tasks, provision of cognitive exercises, or automatic assistance calls in case of a fall of a person. The paper also identified various challenges, including user experience, ethical concerns, and the need for further research to assess long-term acceptance of telepresence robots. The authors suggest that while TPRs show promise in improving the lives of older adults by reducing isolation and enhancing social connections, there are complex issues that need addressing to ensure their effective and ethical use, and long-term positive impact.

Third, Laigaard et al. [33] studied the utilization of TPRs, in the context of urological care. Their study aimed to evaluate the applicability and satisfaction levels of patients, caregivers, and urologists regarding the use of telepresence robots at the urology ward and emergency department. The primary objective was to determine the number of patient encounters that could be resolved without the physical presence of an urologist. The findings showed that in 87% of the encounters, physical presence was not deemed necessary by the participating urologists, suggesting the potential effectiveness of telemedicine in urological care. Despite the challenges in implementing TPRs among urologists, patients expressed their willingness to be attended by TPRs (medical staff mediated through TPRs) in future evaluations, and both patients and caregivers generally reported high satisfaction levels.

Based on the existing literature, especially the works of Wong et al. [31], Isabet et al. [32], and Laigaard et al. [33], we propose three areas in medicine where the use of TPRs could be beneficial to save staff time while maintaining service quality. First, skilled workers can use TPRs to advise less experienced workers, for example when conducting anamnesis. Second, with the help of TPRs, medical professionals could assist people in performing their medical measurements. Third, TPRs could be permanently located in the homes of elderly people to allow their relatives to (remotely) visit them, as well as for medical professionals to give them immediate advice or quickly detect situations that pose a threat to their health. In this way, TPRs could play a vital role in supporting medical interns and offering discreet supervision to elderly patients, facilitating the early detection of critical health disorders.

Based on these notions, we developed three scenarios for using TPRs in medical settings and acted them out in the medical laboratory of Tallinn Health Care College: for designing the scenario involving the use of TPRs in taking anamnesis, the study by Wong et al. [31] served as a guide (*Scenario 1* in Section 3.2); we used the study by Laigaard et al. [33] as a guide for designing the scenario involving TPRs for measuring patients' health data (*Scenario 2* in Section 3.2); and we utilized the study by Isabet et al. [32] as a guide for designing the scenario involving TPRs with elderly individuals (*Scenario 3* in Section 3.2). While designing these scenarios, our goal was to gather understanding if the developed scenarios could be useful in real-life circumstances, and what potential do TPRs have in the medical field from the perspective of medical staff and their educators.

## 3 TPRs in medical settings

In this section, we first describe the telepresence robot used in our studies, followed by the study setup, and details on the study sample and the methods used.

### 3.1 Double 3 TPR

**Fig. 1:**
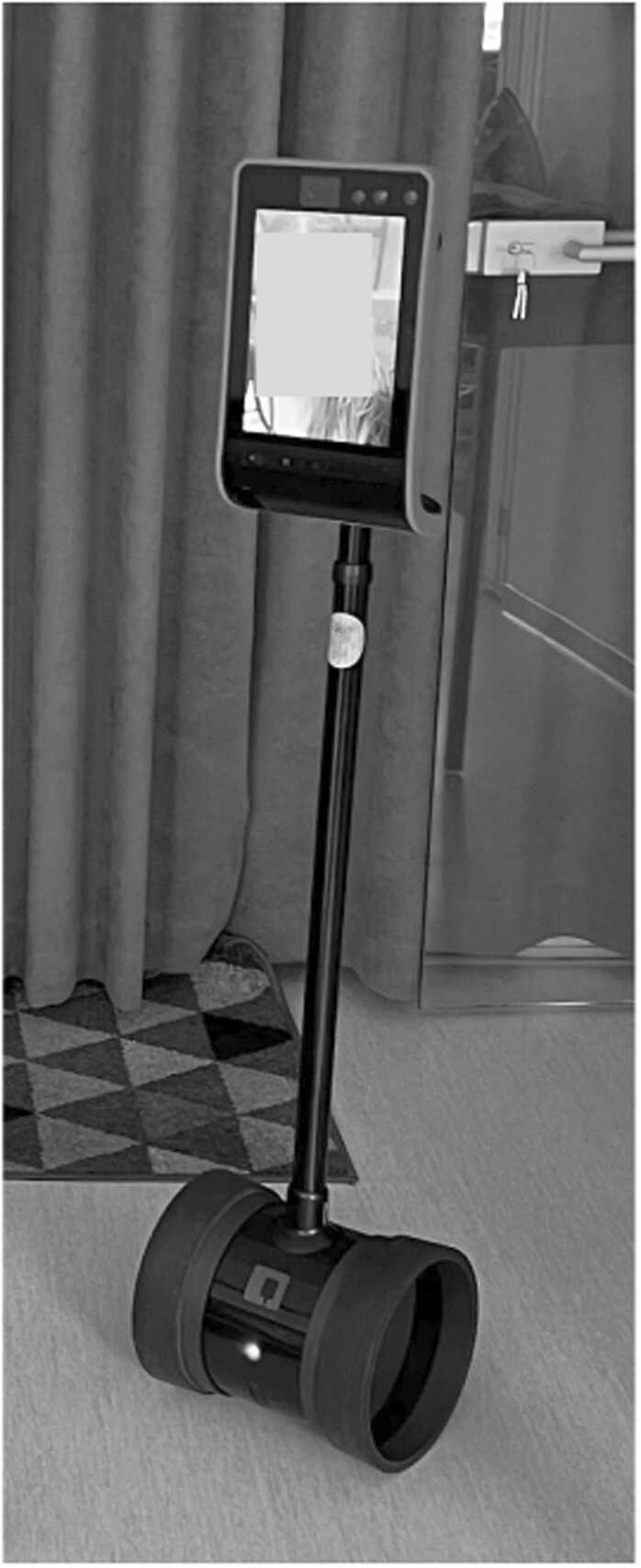
The Double 3 TPR used in the experiments.

For our experiments in three different settings, we utilized the Double 3 TPR – a self-driving, two-wheeled robot (Fig. 1) with the maximum height of 151 cm and the width of 25.4 cm [34]. This robot enables remote individuals to engage with their peers over a distance as if they were physically present. At the top of the robot, a LCD screen displays the remote user’s face and upper body. In addition, the upper part of the robot houses microphones, cameras, and speakers that provide high-quality video and audio for video conferencing, along with various sensors that assist the robot to navigate in its environment and avoid obstacles the remote person might not be able to comprehend. The robot uses Wi-Fi to relay information between its location and the remote person. The remote user can control the robot using either a computer or a smart device. The Double 3 TPR is versatile and can be employed in various settings, such as offices, schools, hospitals, and retail stores, allowing remote individuals to interact with their peers as if they were physically present.

### 3.2 Method and study setup

For the study, we employed an exploratory research approach [35] with qualitative data collection techniques, and tested three TPR usage scenarios in healthcare settings: anamnesis, measurements, and falls and frailty. The study participants (N = 25) comprised teaching staff (18 persons) and administrative employees (1 person) from four higher education institutions: Tallinn University of Technology and Tallinn Health Care College (THCC) from Estonia, and Lappeenranta University of Technology and Jyväskylä University from Finland. In addition the sample included personnel from East-Tallinn Central Hospital (4 persons) and from two private companies, Kinema OÜ and Levira AS (1 person from each). The sample was diverse, with 9 males and 16 females representing various nationalities and ages (23 to 65 years, average age 41). In total, nine participants (36%) of the study had a medical background (5 from THCC and 4 from East-Tallinn Central Hospital).

The individuals included in the study had various experience with semi-autonomous robots, ranging from no prior encounters to brief interactions or even limited utilization in their professional settings. Half of the participants (N = 12) had prior exposure to TPRs. The participants, all of whom maintained cooperative ties with Tallinn University of Technology, received invitations to partake in the experiment through email communication. Participation in the study was voluntary and contingent upon individuals having a personal interest in investigating the behavioral dynamics of semi-autonomous robots, such as TPRs. The experiment was conducted on the June 8, 2023 at THCC, a public Estonian higher education institution offering education and training in the field of health and well-being.

In the context of the experiment, we developed three simulation scenarios that replicate real-world health activities as delineated in Section 2.2: conducting medical histories, performing medical measurements, and visiting older individuals at their residences to identify potential health threats. In addition to the literature reviewed earlier, insights from five participants from THCC with a medical background were incorporated into the scenarios.

**Fig. 2:**
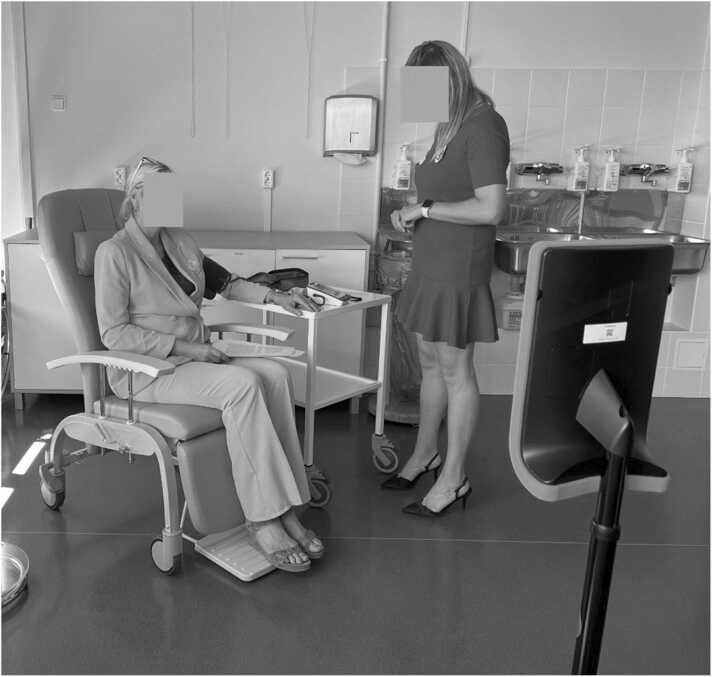
Experiment setup for the 1st scenario – Anamnesis.

*Scenario 1*: Anamnesis (Fig. 2, Appendix A). Here the trainee/student is taking the patient's anamnesis (interviewing the patient about their medical history) for the very first time. The patient is a healthy 40-year-old person undergoing a planned minor operation under local/general anesthesia. The nurse/instructor observes the conversation with the patient through the robot, allowing the trainee/student to practice independently while providing support and guidance as needed.

**Fig. 3:**
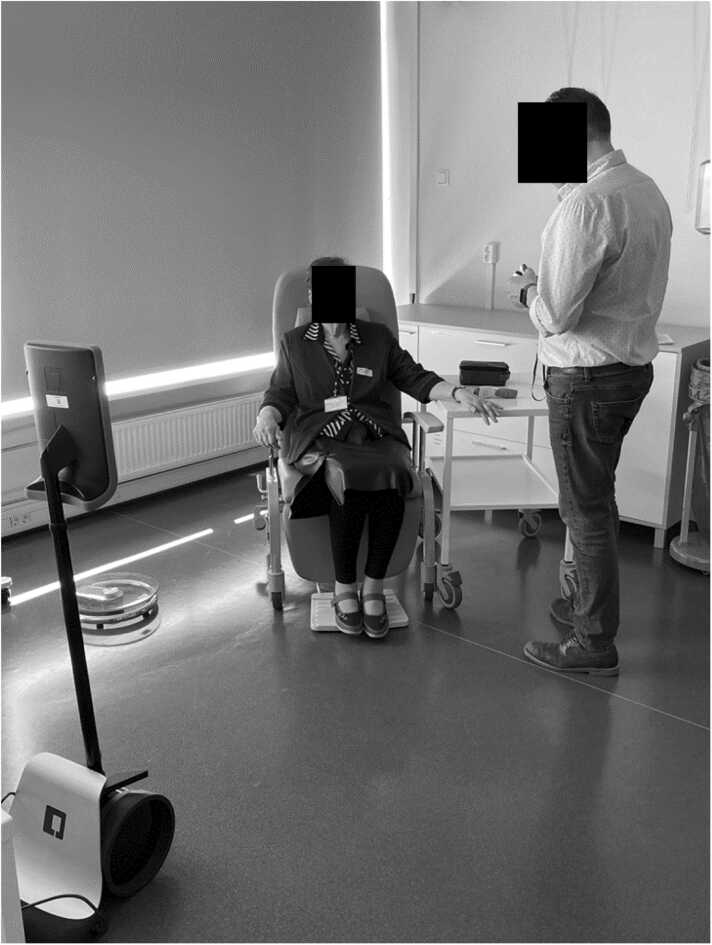
Experiment setup for the 2nd scenario – Measuring Health Data.

*Scenario 2*: Measurements (Fig. 3, Appendix B). Here the intern/learner is going to measure the patient's blood pressure, saturation, body temperature, and weight. The instructing nurse/instructor observes the procedure through the robot to let the intern/learner practice independently while providing guidance when needed. Being in the robot, the instructing nurse/instructor can quickly document the measurement results.

**Fig. 4:**
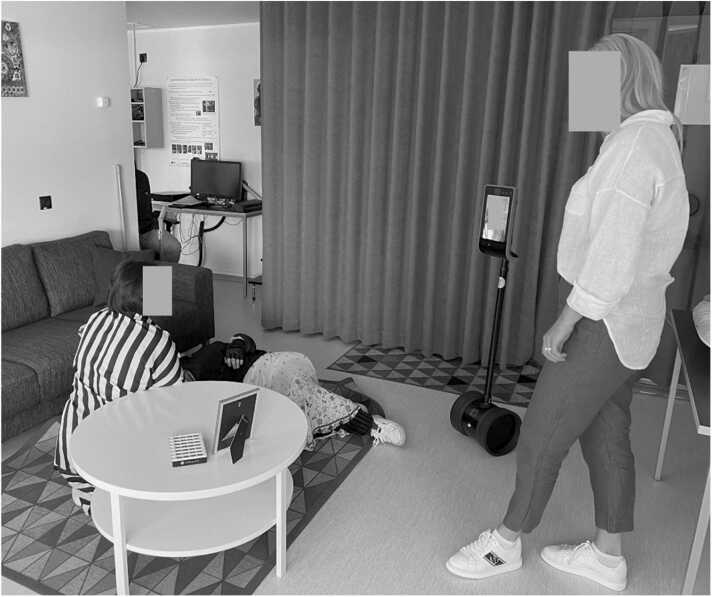
Experiment setup for the 3rd scenario (Falls and Frailty).

*Scenario 3*: Falls and Frailty (Fig. 4, Appendix C). Here, an 85-year-old woman falls in her home and is unable to get up. The 'Falls and Frailty Team' is dispatched to assist the person, provide first aid, and assess whether additional assistance is needed, such as transporting her to the hospital or determining if home modifications are necessary.

With the exception of five participants from THCC with a medical background, the remaining participants were deliberately kept uninformed of the scenarios and unprepared for the experiment. Before starting the experiment, participants were briefed on the specific scenarios, and assigned distinct roles within the scenarios. The five participants from THCC actively participated in the experiment, with two in Scenarios 1 and 2, and one in Scenario 3. They undertook medical roles such as occupational therapists, occupational therapy interns, and paramedics, using TPRs to tend to physically ill patients. All other study participants took roles, as either patients, patients' relatives, or assisting staff such as paramedics. It was ensured that each participant had an opportunity to engage actively in the simulations. Each experiment lasted for up to about 30 min, after which participants who were not from THCC proceeded to another experiment with a different scenario, while the participants from THCC remained in the room of their original experiment. If the room of the next experiment was occupied then the participants waited in the rest area and could interact with each other.

Subsequent to the experiment, semi-structured online interviews were conducted with the five medical field employees of THCC (four females and one male, age ranging from 35 to 75 years, with the average age of 52 years). During these interviews, the interviewed participants were requested to assess the strengths, weaknesses, and practical applicability of TPRs in real-life medical situations simulated by the scenarios. The interviews were conducted and recorded by one of the article author at the participants' workplace two weeks after the experiment to allow interviewees more deeply understand the viability aspects of using TPRs in health care. Transcriptions of these interviews were carried out using Microsoft Word 365 and were openly coded by two researchers.

### 3.3 Ethics statement

Written consent was obtained from all participants before the experiment. The participants signed a consent form, which indicated that personalized data would not be collected. The form also specified that faces in all photos would be blurred for research purposes, and videos would be deleted after analyzing observational data. In accordance with national laws and the regulations of the authors' universities, the study did not require ethics committee approval as it did not qualify as a 'human study' according to the definitions outlined in the Declaration of Helsinki (1964, 2008). The study did not involve clinical research or a vulnerable population (all participants were healthy adults), and it did not include identifiable human data. The research involved observing human behavior in the form of 'Benign Behavioral Interventions with Adults' and excluded minors. The interventions were brief, harmless, painless, and non-physically invasive. They were not offensive or embarrassing, did not involve deception, and did not result in any lasting significant adverse impacts on the participants.

## 4 Results

The aim of our research was to investigate the potential of TPR applicability in healthcare through developing and evaluating three scenarios (Section 3.2) for using TPRs in health care situations where the use of TPRs could reduce the workload of medical personnel or attend persons while physical presence is limited.

### 4.1 TPR experience assessment

Practically all (4) interviewed persons found in *expressis verbis* that trying out our scenarios with TPRs was a novel, exciting and positive experience (I1: “*Quite a new experience. for our school. for the employees, both for those who participated and for those who watched. Overall, it was very exciting.*”) From the point of view of health care teaching, TPRs were found to be a feasible technological learning tool to be integrated into teaching and learning (I3: “*For me, any innovation related to learning and teaching is quite important, and it must be as simple as possible while providing a lot to the learner.*”), to critically discuss various health care situations (I3: “*To go through situations with students so that critical thinking is developed in them through these situations.*”) Moreover, the technology was seen as a promising way to save workers' time while at the same time preventing the spread of infectious diseases (I4: “*These topics are all interconnected when it comes to infections. It helps save human factor costs, prevent the spread of certain bad things, and reduce wear and tear, as well as work-related stress.*”).

The interviewees suggested that TPRs could really present persons in interaction (I1: “*The entire situation essentially demonstrated that, indeed, a robot can represent a human.*”), i.e., provide remote persons with better social presence (I3: “*Because I'm actually communicating with someone. I see the person, and I see what they are doing. So what if there's a screen in between us? I consider it as a form of contact, and for the patient, human contact is still very important. If that touch is missing from the equation, it might not be that significant*.”) However, the social presence provided is still weaker than that of real physical presence (I3: “*Empathy and support are one of my strengths as a nurse. We should be by the patient's side, and I mean it quite literally. Because, in certain situations, it's not just about our voice and guidance; physical contact is more important,*”) and this delicate balance seems to be one the keys to defining the user experience of TPRs in our scenarios.

### 4.2 Usefulness of the proposed scenarios

Next, we analyzed the usefulness of the proposed scenarios based on the feedback collected from the interviews. All participants found that in all three scenarios TPRs could be extremely useful when physical isolation is unavoidable in order to ensure sterility of the room or to prevent the spread of infectious diseases (I5: “*With airborne infectious diseases, like if you don't want tuberculosis, which’ open form is quite dangerous, (using a robot) to deliver or retrieve something there. Likewise, in the case of the children's hospital section, if a parent can't be with their child, I would invite them to be present through the robot during a procedure.*”; and I3 “*Whether it's in a sterile environment or there's a risk of infection, in this case, I can indeed see the involvement of such a robot, assuming, of course, that the robot is already inside that room.*”).

However, several contraindications were brought out for the situations where the conditions do not justify physical isolation. For example, some people could be negatively biased towards replacing a direct physical presence of nurses or doctors with a TPR-mediated presence (I4: “*Again, the same arguments for and against still remain, as some people prefer humans.*”), communication via the robot could be difficult for people with hard hearing (I2: “*Right, our table and the robot weren't the only ones. There was also that lady from another table who said, 'Oh, I can't hear them at all!*'”) In addition, for taking measurements, it was pointed out that some of these procedures need to be physically demonstrated in order to ensure correct results (I5: “*In fact, the same goes, for example, I start guiding you through Zoom. And you show me. Here you need to place your hand in the right position and in the right place so that, for example, when we measure pressure and so on. Through the camera, it's somehow a 'no' for me.*”).

A few of the interviewees pointed out that the technology of TPRs while promising, is still in early development stages and thus has its limitations, such as the inability to actually lift fallen persons (I5: “"*Informs me that the patient has fallen; the robot cannot assist you with this matter.*”) or move around in people’s homes (I3: “*Can this robot move around there without obstacles, whether it's a carpet, furniture, a high door frame, or anything else?*”). For these reasons, the third scenario (“Falls and frailty”) for using TPRs, while considered realistic by some interviewees, was considered impractical in the real-world conditions by others. Similarly, the interviewees drew attention to the need to test TPRs in less idealistic real-world conditions to get a better understanding about their real usefulness (I4: “*Let's say that when conducted in a controlled laboratory setting, everything could work, but what interests me more is actual, real-world testing on real patients or a real target group. It's from there that things can emerge, and it will be revealed whether people find these things unfamiliar or how they adapt to them in a home setting, as I do as a nurse.*”).

In summary, the study/interviews???? Indicated that although TRPs are seen as novel and potentially revolutionizing technology for healthcare, there exists a strong need for further and deeper studies under real-life conditions before TPR technology can be adopted for real medical cases.

## 5 Discussion and conclusions

The ageing population presents a challenge to the healthcare sector workforce's capacity to deliver sufficient services to their patients. One potential solution to enhance the capacity of healthcare professionals is the utilization of telepresence robots (TPRs), which can expedite and cost-effectively extend medical assistance to a larger number of individuals in need of care. In this study, we conducted a comprehensive review of the existing literature on the utilization of TPRs in healthcare. Drawing inspiration from the literature, we designed three potential usage scenarios for TPRs in medical settings, which were subsequently tested in the laboratory of Tallinn Health Care College. We collected feedback data from five medical field professionals of the same institution, all of whom took part in the experiment.

Our findings reveal that individuals across various age groups and genders exhibited a general willingness to engage with emerging technologies like TPRs within their professional domains. Furthermore, this technology garnered a widespread perception of promise and utility. Nevertheless, it became evident that the applicability of TPRs may be constrained by their current technical limitations, including challenges related to traversing physical thresholds and audio quality deficiencies. The potential resistance from patients should also be considered, highlighting the need for a judicious approach to TPR deployment, particularly in sensitive healthcare contexts. These implications align with the observations presented in [35].

However, it is worth noting that TPRs show promise in enhancing the preparedness of healthcare workers for future technologies, as also indicated in [36] and [37]. The latter underscores the potential of TPRs as educational tools within the healthcare sector. Moreover, in situations where physical isolation was deemed necessary, TPRs were regarded as viable alternatives for augmenting the social presence of medical personnel, with the potential to enhance the mental health and well-being of patients. This observation aligns with the findings in [28] and [38].

To investigate the potential applicability of TPR in healthcare, we formulated three distinct usage scenarios: (1) Anamnesis (Appendix A); (2) Measurements (Appendix B); and (3) Falls and Frailty (Appendix C). Our study's findings suggest that, while direct physical interaction with healthcare professionals is always preferable, these scenarios remain applicable in real-world contexts when personnel time is limited or when the risk of contagion is high. For instance, the authors of [39] have proposed that telepresence robotic assistants can be effectively utilized in the early triage phases, significantly mitigating the risk of contagion, thus corroborating our first scenario. In alignment with our second scenario, the authors of [33] have indicated that TPRs can be employed to assist patients in self-administering health measurements in specific domains. In our experimental approach to our third scenario, the robots we had chosen were not equipped to aid elderly individuals in standing up after a fall but were rather used as local agents to identify health-critical situations. The latter limitation could potentially be addressed by employing more advanced robots, such as the RIBA robot discussed in [40]. For all tested scenarios, it was indicated by interviewees that TPRs in their present form and functionality have limitations, restricting their successful adoption. However, as pointed out in [41], short malfunctioning of TPRs does not present a threat to human life and health, whereas TPRs, in general, can offer support and safety to the patients. In addition, drawing on recent developments in AI technologies [42], it could be reasonable to suggest that in the future, AI-backed robot assistants could act independently in scenarios described in this study.

In conclusion, our findings suggest that, in principle, the three healthcare scenarios we proposed were successful and showed the potential effective use of TPRs in healthcare. However, further research needs to be warranted to conduct more comprehensive studies testing these scenarios in real-life conditions.

### 5.1 Limitations and future directions

The experiments were conducted in a controlled laboratory environment, offering insights into the potential of TPRs. However, these simulations fall short of replicating the real-world complexity of healthcare contexts, which involves time-sensitive activities and potential ethical and privacy concerns related to TPRs' cameras, microphones, image data, and surveillance implications. Furthermore, the study's relatively small sample size may limit the generalizability of its findings. Therefore, additional research is necessary to test TPRs in authentic healthcare environments and address these limitations, ensuring their effective and ethical integration into elderly healthcare.

Our research presented scenario-specific findings, focusing on the utility of TPRs in distinct healthcare situations. It is crucial to acknowledge that the applicability of TPRs may vary across diverse medical contexts. Ethical and privacy concerns associated with TPR usage were not extensively explored, leaving these critical considerations for future implementations to address. In summary, further comprehensive research is required to evaluate TPRs in real healthcare settings and overcome these limitations for their effective and ethical integration into elderly healthcare.

In terms of future work, we have planned to elaborate the proposed scenarios and conduct these experiments in real-life situations, explore the patient side of TPR applicability in medical settings, including potential patient resistance, and investing the applicability of TPRs for medical education.

## Declaration of Competing Interest

The authors declare that they have no known competing financial interests or personal relationships that could have appeared to influence the work reported in this paper.

## Appendix A Anamnesis

**Approximate duration**: 15 min.

**Participants**: Double 3 TRP (1) - Nurse/Instructor (1 participant) - Trainee/Student (1 participant) - Patient (1 participant) - Remaining observers.

**Room**: One private room for collecting anamnesis. 1 chair for the patient to sit on, 1 chair for the trainee/student. The participant playing the role of the nurse/instructor guiding the trainee/student through the TPR sits at a table, using a computer connected to a projector in the room to allow other participants to see the robot's perspective. Observers can sit on chairs if desired.

**Equipment**: Robot, computer, headphones, participants, paper/anamnesis form, and pencil.

**Scenario description**: The situation takes place in a private room where the trainee/student is taking the patient's anamnesis (interviewing the patient about their medical history) for the very first time. The patient is a healthy 40-year-old person undergoing a planned minor operation under local/general anesthesia. The nurse/instructor observes the conversation with the patient through the robot, allowing the trainee/student to practice independently while providing support and guidance as needed.

**[Scene 1: Introduction and preparations]**.

The nurse/instructor logs into the appropriate environment through the computer and establishes a connection with the robot. Before approaching the patient, the trainee/student receives advice from the instructor via the robot on how to take anamnesis, including nuances of formulating questions and the importance of active listening.

**[Scene 2: Collecting anamnesis]**.

**Arriving at the patient and explaining the situation**: The trainee/student invites the patient to accompany them to a suitable secluded room for taking anamnesis, greets the patient, introduces themselves, and explains that the patient needs to answer some health-related questions before the operation. The nurse/instructor drives the robot to the patient and trainee/student, greets and introduces themselves through the robot, explains the purpose of their presence through the robot, and asks if the patient agrees with it.

**Creating a suitable environment**: The nurse/instructor moves the robot to a suitable position where there is good visibility for both parties and ensures that they are visible and audible as well. The trainee/student asks the patient to sit and takes a seat opposite them.

**The interview begins**: The trainee/student asks the patient about their general health and previous surgeries.

For example: Starts with patient identification, asking for first and last name and date of birth.

• How would you assess your overall health?
• Have you had any significant health problems or chronic illnesses in the past?
• Are you currently taking any medications or supplements?
• Do you have any known allergies to medications, food, or other substances?
• Have you ever been hospitalized or had any previous surgeries?
• Do you currently have any symptoms or complaints that could indicate a health problem, such as changes in energy level or appetite?
• Do you engage in regular exercise or physical activity? If yes, could you please describe the type and frequency?
• How would you describe your typical daily diet?
• Do you have any specific dietary restrictions or preferences?
• Do you smoke or consume alcohol? If yes, how much, how often, and how many units/pieces per day?

**During the interview**: The nurse/instructor encourages the trainee/student, through the robot, to ask additional questions, such as "Could you please clarify?" "Can you name?" "Please provide more details." If necessary, the nurse/instructor adjusts the question structure to encourage asking open-ended questions. They ensure that all essential questions are asked and gather parallel information for developing a nursing care plan.

**At the end of the interview**: The trainee/student and nurse/instructor praise and thank the patient.

Then it is explained that the next step is to measure their blood pressure, saturation, body temperature, and weight.

The nurse/instructor acknowledges the trainee/student for their excellent work and provides recommendations for the future if necessary.

## Appendix B Measurements

**Approximate time:** 15–20 min.

**Participants:** Double D3 TRP (1) - Nurse/Instructor (1 participant) - Intern/Student (1 participant) - Patient (1 participant) - Other observers.

**Location:** One hospital room with beds, 1 bed available for the participant playing the patient. 1 chair next to the bed for the participant playing the intern/learner. The participant playing the instructing nurse/instructor is behind a computer connected to a television in the room, allowing other participants to see the robot's perspective. Observers can sit.

**Equipment:** Robot, computer, participants, blood pressure monitor, thermometer, scale, and pulse oximeter.

**Scenario Description:** The situation takes place in a ward where the intern/learner is going to measure the patient's blood pressure, saturation, body temperature, and weight. The instructing nurse/instructor observes the procedure through the robot to let the intern/learner practice independently while providing guidance when needed. Being in the robot, the instructing nurse/instructor can quickly document the measurement results.

**[Scene 1: Introduction]**.

The intern/learner has prepared all the necessary equipment and signals to the instructor that they are ready for the measurements, requesting guidance throughout the process. The instructing nurse/instructor is ready to assist at every step.

**[Scene 2: Performing the Measurements]**.

The instructing nurse/instructor moves towards the patient in the robot. The patient is comfortably seated on the bed, prepared for the measurements. Then, the instructing nurse/instructor positions themselves according to the location chosen by the intern/learner to have the best possible overview of the procedures.

The intern/learner explains to the patient that they will start by measuring their blood pressure. The instructing nurse/instructor provides instructions on the correct placement of the cuff and how to use the device. The intern/learner follows the instructions and begins the measurement. They relay the results to the instructing nurse/instructor, who immediately records them in the documentation. The instructor comments that everything is within the normal range, and the cuff can be removed. The intern/learner praises the patient.

Next, the intern/learner measures the pulse and oxygen saturation by placing a pulse oximeter on the patient's finger. The instructing nurse/instructor advises on the proper positioning and what to observe. The intern/learner relays the pulse and oxygen saturation readings, and the instructor records them. The intern/learner removes the device and informs the patient that the measurements are within the normal range.

Then, the intern/learner measures the patient's body temperature. The instructor guides them on how to turn on the thermometer and the appropriate distance to hold the non-contact thermometer. Again, the measurements are recorded, and the results are shared with the patient.

Lastly, the intern/learner weighs the patient by asking them to step on the scale, and the result is documented. The patient is praised and thanked for their cooperation.

The instructing nurse/instructor commends the intern/learner for their excellent work and provides recommendations if necessary for future practice.

## Appendix C Falls and frailty

**Participants**: Double 3 TRP (1) (Occupational therapist through a telepresence robot, 1 paramedic, 1 occupational therapy intern), 85-year-old woman.

**Setting**: 85-year-old woman's home (kitchen, living room, bathroom).

**Scenario Description**: An 85-year-old woman falls in her home and is unable to get up. The "Falls and Frailty Team" is dispatched to assist the woman, provide first aid, and assess the need for additional assistance (whether to transport her to the hospital or if home modifications are necessary).

**Introduction**: Emergency services receive a call from an 85-year-old woman who has fallen in her living room and cannot get up. The specialized "Falls and Frailty Team" is dispatched, typically consisting of an occupational therapist and a paramedic, aiming to prevent hospitalization of fallen individuals by providing initial home assessment and assistance. Due to a shortage of occupational therapists and long distances, the occupational therapist participates remotely using a telepresence robot. The robot is provided by the emergency services (i.e., the paramedic). The occupational therapist is accompanied by an intern on these visits. The paramedic has entered the woman's home, and her condition is stable, while the occupational therapist joins as a telepresence robot.

**Content**: The paramedic ensures the woman's stable condition. Together with the intern, they help her sit up, provide first aid for scraped knees and arm. The occupational therapist observes the process. The cause of the fall is identified. The woman is allowed to move around the room (occasionally relying on objects for support, confirming it feels safer when asked) and does not use any assistive devices. The occupational therapist and the intern familiarize themselves with the woman's home and conduct an initial assessment of the home environment (checking for bath/shower accessibility, determining the need for handrails or raised toilet seats, identifying loose carpets, etc.). It is collectively decided that the woman could benefit from a walker (the team's vehicle carries a selection of wheelchairs, walkers, and canes), which is tried out with her.

**Summary**: Since the woman does not require hospitalization, an additional visit is arranged by the occupational therapist and a home modification specialist to implement necessary adjustments and teach safer transfer techniques.
